# Emerging Roles of SRSF3 as a Therapeutic Target for Cancer

**DOI:** 10.3389/fonc.2020.577636

**Published:** 2020-09-25

**Authors:** Zhixia Zhou, Qi Gong, Zhijuan Lin, Yin Wang, Mengkun Li, Lu Wang, Hongfei Ding, Peifeng Li

**Affiliations:** ^1^Institute for Translational Medicine, The Affiliated Hospital of Qingdao University, College of Medicine, Qingdao University, Qingdao, China; ^2^Departments of Pediatrics, Second Clinical Medical College of Qingdao University, Qingdao, China; ^3^Key Laboratory for Immunology in Universities of Shandong Province, School of Clinical Medicine, Weifang Medical University, Weifang, China

**Keywords:** SRSF3, oncogene, RNA splicing, cancer, alternative splicing

## Abstract

Ser/Arg-rich (SR) proteins are RNA-binding proteins known as constitutive and alternative splicing (AS) regulators that regulate multiple aspects of the gene expression program. Ser/Arg-rich splicing factor 3 (SRSF3) is the smallest member of the SR protein family, and its level is controlled by multiple factors and involves complex mechanisms in eukaryote cells, whereas the aberrant expression of SRSF3 is associated with many human diseases, including cancer. Here, we review state-of-the-art research on SRSF3 in terms of its function, expression, and misregulation in human cancers. We emphasize the negative consequences of the overexpression of the SRSF3 oncogene in cancers, the pathways underlying SRSF3-mediated transformation, and implications of potential anticancer drugs by downregulation of SRSF3 expression for cancer therapy. Cumulative research on SRSF3 provides critical insight into its essential part in maintaining cellular processes, offering potential new targets for anti-cancer therapy.

## Background

**Figure 1:**
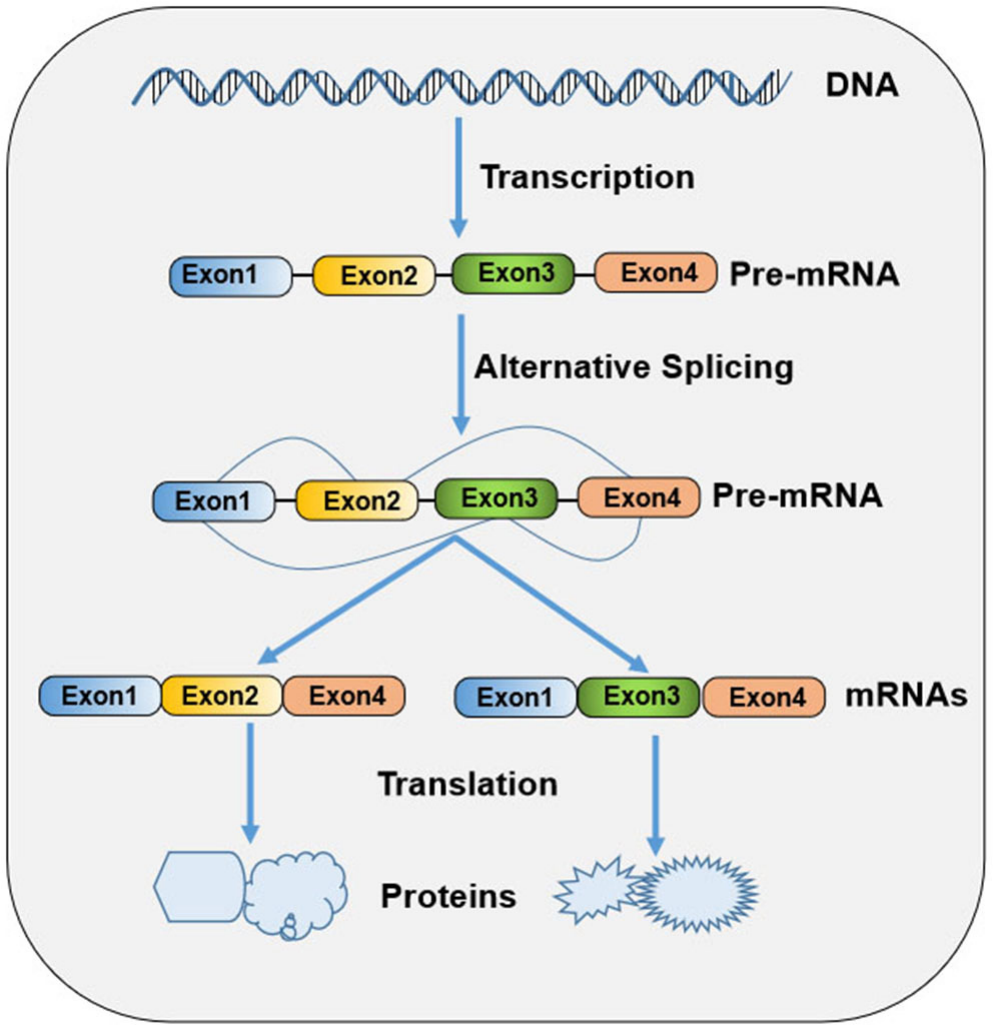
Schematic diagram of gene expression in prokaryote cells.

Ribonucleic acid (RNA) splicing is a fundamental process of gene expression, during which non-coding sequences (introns) are removed and coding sequences (exons) are ligated together from a precursor messenger RNA (pre-mRNA) to form a mature messenger RNA (mRNA) (1). In higher eukaryotes, most genes undergo alternative splicing from a single pre-mRNA transcript via splice site selection, generating multiple mature mRNAs that have different functions and contribute to biologic complexity (Figure 1). Both constitutive and alternative splicing processes are catalyzed by dynamic and complex macromolecular major (U2-dependent) or minor (U12-dependent) spliceosomes (2). Each spliceosome contains five small nuclear ribonucleoprotein (snRNPs) particles: U1, U2, U4, U5, and U6 snRNAs for former and U11, U12, U4atac, U5, and U6atac snRNAs for the latter (3). Spliceosome recognizes the consensus sequence elements at the 5'ss, 3'ss, and branch point (BP) sites, which is a crucial step in the splicing pathway (3). The selection of splice sites for recognition is modulated by an array of RNA regulatory sequence elements, including exonic and intronic splicing enhancers and silencers. These splicing regulatory elements (SREs) are recognized by numerous accessory splicing factors, including the heterogeneous nuclear ribonucleoproteins (hnRNPs) and Ser/Arg-rich (SR) proteins (3). SR proteins and hnRNPs promote and suppress splicing, respectively, in a sequence-depending manner and in diverse ways, including facilitating the recruitment of U1 or U2 snRNP, occluding a splice site, and “looping out” an exon (4). In addition to their role as splicing regulators, these proteins also participate in other diverse RNA metabolic processes and cellular processes, such as Pol II transcription, mRNA export and translation, genomic stability maintenance, cell viability, and cell-cycle progression (5).

An increasing body of evidence supports that the aberrant splicing of pre-mRNA results in the production of aberrant proteins that contribute to the misregulation of cellular growth, differentiation, and tissue development, eventually leading to the susceptibility to diseases, including cancer (6). Recent studies have found that alterations and mutations in the genes encoding core spliceosomal proteins and related RNA-splicing factors provide major mechanisms for cancer-associated splicing and transformation, implicating tumor establishment, progression, and resistance to therapy (4, 6). Many splicing factors, including SR and hnRNP families, have been certified to act as both oncoproteins (or proto-oncoproteins) and tumor suppressors. Therefore, we focus on Ser/Arg-rich splicing factor 3 (SRSF3), also called SRp20, which is a member of the highly conserved SR protein family. SRSF3 plays a critical role in the regulation of RNA splicing and many other cellular functions. Aberrant SRSF3 function can be identified in several human diseases, including Alzheimer's disease (7), systolic heart failure (8), ocular hypertension (9), virus infection (10–12), and tumor (13). In this review, we summarize current research on the function and expression regulation of SRSF3 and the misregulation and biological implications of SRSF3 in cancer, as well as its therapeutic potential.

## SRSF3 Functions

SR protein family are identified by possessing one or two N-terminal RNA-recognition motif (RRM) domains and a C-terminal domain enriched with the Arginine (R) and Serine (S) amino acid sequences (RS domain). In general terms, RRM domains recognize RNA, whereas RS domains take part in diverse protein-protein and protein-RNA interactions (14, 15). Thus, far, 12 members of the SR protein family have been identified in humans, encoded by 12 genes and designated SRSF1-12. All members of the SR protein family are mainly nuclear and localize to interchromatin granule clusters (IGCs) or nuclear speckles, but some members including SRSF3 can shuttle between the nucleus and the cytoplasm (15–17). SR proteins have been shown to regulate constitutive and alternative splicing as well as multiple other steps of RNA biological metabolism, suggesting that they are multifunctional proteins taking part in transcriptional, co-transcriptional, and post-transcriptional regulation pathways (18, 19). Given the important roles that SR proteins act on these processes, aberrant expression and/or activation and somatic mutation in SR proteins would lead to developmental impairments and disease pathophysiology (4, 20).

**Figure 2:**
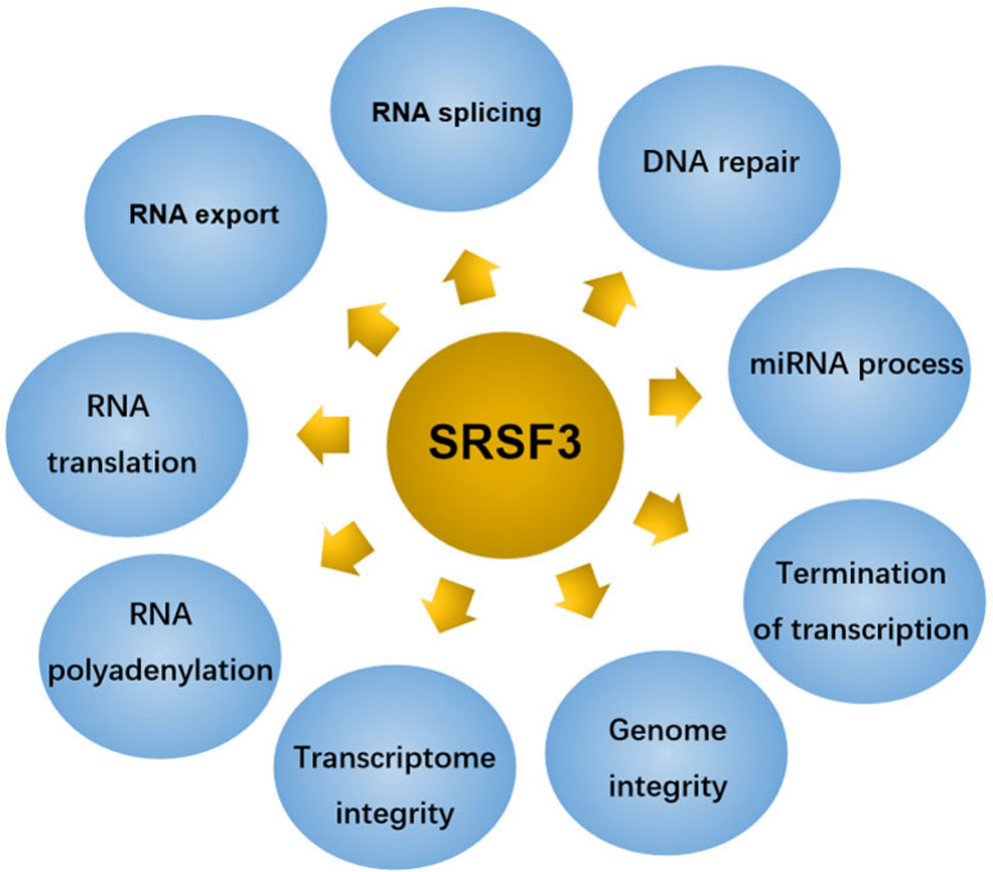
SRSF3 regulates several cellular functions in eukaryote cells.

SRSF3 composes 164 amino acids with 19 kDa molecular weight makes it to be the smallest member of the SR protein family (13). Although initially identified as a splicing regulator, SRSF3 has been identified as a polyfunctional protein involved in multiple physiological and pathological processes, as shown in Figure 2.

### Regulation of Splicing

SRSF3 regulates the global change in gene expression program to maintain cell homeostasis by constitutive splicing and alternative splicing (21). Alternative splicing is an essential process for regulating most protein-coding genes by producing multiform messenger RNA transcripts to yield proteomic diversity in eukaryotic cells. Several distinct patterns of SRSF3-induced alternative splicing exist, including mutually exclusive exons (22), alternative terminal exons (23), alternative cassette exons (where one or more exons is either skipped or included) (24–26), alternative unique exon (27), skipping of 5'-nucleotides from exon (28), intron retention (IR) (29, 30), and early termination codon (30). In addition, SRSF3 can act as activators or repressors in the alternative splicing of other RNA-binding proteins (RBPs), such as SRSF1 (31), SRSF5 (32), C-terminal domain (CTD) of RNA polymerase II (pol II) (25), polypyrimidine tract-binding protein (PTBP) 1 and heterogeneous nuclear ribonucleoprotein (hnRNPs) A1 (33). Similar to other SR members, SRSF3-related aberrant splicing is often associated with the non-sense-mediated mRNA decay pathway, resulting in inducing aberrant protein isoforms that are often linked to numerous human diseases, including cancers.

### Regulation of RNA Export

The ability of SRSF3 to shuttle between the nucleus and the cytoplasm allows it to be a major contributor to the regulation of mRNA export. Similar to other SRSFs, the nuclear to cytoplasmic translocation of SRSF3 relates to the export receptor, nuclear export factor 1 (NXF1) via Arginine-rich peptide adjacent to RRM (34). Among those SRSFs, SRSF3 presents the most effective adaptor for the NXF1 adaptor (35), suggesting that SRSF3 can act as a sturdy ship in the TAP-dependent mRNA export from nucleus to cytoplasm. SRSF3 represses nuclear export of programmed cell death (*PDCD4*), isoform 2 mRNA. Consequently, at SRSF3 knockdown, *PDCD4 AS-2* mRNA level, but not *AS-1*, was found to increase in the cytoplasm (36). In addition, SRSF3 can interact with NXF1-nuclear transport factor 2-related export protein 1 (NXF1-NXT1), resulting in the export of “intronless” mRNAs (37).

### Regulation of RNA Translation

SRSF3 can mediate post-transcriptional regulation of mRNA. It presents an internal ribosome entry site (IRES) and mediates the translation initiation of viral RNA in company with PCBP2, an IRES-binding protein (38). Moreover, it was reported that SRSF3 is not only participating in pre-mRNA alternative splicing but also in the regulation of the translation of *PDCD4* mRNA. Of the two alternatively spliced transcripts of *PDCD4*, only isoform 1 (the major isoform) was found to be affected at the translational level by SRSF3. Further study found that SRSF3 exerted its effect on *PDCD4* mRNA translation through a strong interaction with the 5'-untranslated region (5'-UTR) and recruitment to P-bodys (PBs). When SRSF3 was silencing, PBs disappeared and the translation inhibition of *PDCD4* mRNA was relieved. These data investigate that SRSF3 recruits *PDCD4* mRNA to PBs for the expression of PDCD4 (36, 39). In addition, a reproducible hypoxia-induced increase in SRSF3 protein was associated with the hypoxic stress-induced retained intron (RI) in translation initiation of *EIF2B5* (29). RI in EIF2B5 creates a premature termination codon (PTC), leading to a 65kDa truncated protein isoform that opposes full-length eIF2Bε to inhibit global translation. Upon SRSF3 knockdown, the expression of the 65 kDa isoform of eIF2Bε disappearances in normoxia or hypoxia conditions. Then the biding between SRSF3 protein and *EIF2B5* mRNA was proved to increased (29). These results indicate SRSF3 as a regulator mediating RI in EIF2B5, consequently taking part in translational control under hypoxia. Moreover, SRSF3 was identified as a translation regulator of innate immune genes, which may be because there are several putative binding sites for SRSF3 in 3′ UTRs of some innate immune gene (40). As expected, SRSF3 silencing led to the increase in the protein synthesis of immune mediators, containing SAA3, CCL5, and CCL3, suggesting that SRSF3-mediated translational regulation is involved in innate immunity (40).

### Regulation of RNA Polyadenylation

Polyadenylation is a processing step for generating mature mRNA in eukaryotes (41). In the model for the negative regulator of splicing (NRS)-stimulated Rous sarcoma virus (RSV) polyadenylation, it was shown that SR proteins, including ASF/SF2, 9G8, and SRSF3, binding to NRS- or systematic evolution of ligands by exponential enrichment (SELEX)-binding sites was sufficient to stimulate polyadenylation *in vitro*. However, just SR protein-binding sites promoted polyadenylation independent of the NRS complex *in vivo* when moved nearer to the viral poly(A) site. Data manifest that SR proteins play a promoting role in RSV polyadenylation, but only when they are close to the RNA 3′ end by binding to the NRS (42). In addition, SRSF3 was reported to affect the recognition of an alternative 3′-terminal exon by effecting the efficiency with which a polyadenylation factor is bound to an alternative polyadenylation site (43). These results suggest that SR proteins not only regulators the polyadenylation of cellular mRNAs but also controls alternative polyadenylation.

### Regulation of Transcriptome Integrity

SRSF3 is also reported to contribute to the establishment and modulation of the maternal transcriptome (19). SRSF3 was proved to highly express in germinal vesicle (GV) and MII oocytes (at metaphase of meiosis II), indicating SRSF3 acting as a critical maternally inherited factor. SRSF3 knockdown in grown germinal vesicle oocytes compromises the capacity of germinal vesicle breakdown (GVBD). Further, the GVBD defect in mutant oocytes was proved to be due to both aberrant alternative splicing (including *Brd8* and *Pdlim7*) and depression of B2 SINE transposable elements. These observations suggest that the control of the transcriptional identity of the maternal transcriptome by SRSF3 is essential to the development of fertilized competent oocytes (19).

### Regulation of Genome Integrity

DNA lesions are usually caused by chemical compounds with (pro-)genotoxic activity and dysregulations of basic processes, including transcription, DNA replication, and mitosis (44). Mitotic distortions and transcription-associated RNA-DNA hybrids (R-loops) formations induced by impaired expression of RNA-binding proteins are strongly associated with DNA injury (45). SLU7 as a key mediator of genome stability was reported to be required for the mitotic progression of transformed cells, suitable spindle assembly, sister chromatid cohesion (SCC), and sororin splicing regulation, as well as for the protecting cells from R-loop formation and DNA damage (45). SLU7 knockdown leads to the formation of R-loops, DNA damage, cell-cycle arrest, and SCC loss. Further study found that SLU7 regulates the splicing of *SRSF1* and *SRSF3* and inhibites the protein expression of truncated SRSF3 (SRSF3-TR) (45). These results demonstrate that SRSF3-TR proteins, as a target of SLU7, may play a important role in DNA damage and genome instability.

### Regulation of Transcription Termination

Termination of RNA polymerase II (Pol II)-mediated transcription acts a significant role in the regulation of gene expression (46). The transcription termination of RNA polymerase II (Pol II) contains two linked steps: mRNA 3′-end formation and Pol II release from DNA. The intact 3′-processing signal and some 3′-end processing factors are also required for Pol II transcription termination (47, 48). In the model of a *C. elegans* operon intended to select factors taking part in the transcription termination, the lin-15 operon involves two genes: *lin-15B* and *lin-l5A* (49). Two deletion alleles of *rsp-6*, which encodes SRSF3, were found to strongly suppress the synthetic Multivulva phenotype of *lin-15AB* (n765) at levels similar to RNAi. In *lin-15AB*, RNA levels decrease markely at the site of the insertion, whereas they restore at the site of the insertion in the *rsp-6* mutant strain. Further, SRSF3 was found to increase the RNA downstream of the cleavage site without influencing cleavage (49). These data indicate that SRSF3 acts a role in termination of transcription and not in cleavage, maybe by interacting with the RNA downstream of the cleavage site.

### Regulation of miRNA Process

SRSF3 was also demonstrated to facilitate primary microRNA transcripts (pri-miRNAs) recognition and processing. Pri-miRNAs own at least one RNA motif in the major and conserved motif family: UG, UGU, and CNNC (50). The UG and UGU motifs of pri-miRNAs cooperate with the microprocessor complex (including RNase III DROSHA and DGCR8 dimer) to cleave pri-miRNAs to initiate microRNA (miRNA) maturation, whereas CNNC connects with SRSF3 to induce the microprocessor to process pri-miRNAs. That is, SRSF3 supplies DROSHA to the foundational junction in a CNNC-dependent manner, then improving microprocessor activity (50). For example, a genetic variant (G27-to-A variant) in the terminal loop (*TL*) of pri-mir-30c-1 leads to the reorganization of the RNA secondary structure, thereby promotes the interaction of pri-mir-30c-1 with SRSF3. And the interaction between them occurs at the CNNC motif located 17 nucleotides away from the Drosha cleavage site at the basal region of the G/A variant. This interaction, also increases the microprocessor-mediated processing of primir-30c-1, causing the upregulation of miR-30c level (51). In addition, expressions of mature miR-1908-5p (52) and miR-3131 (53) were also mediated by SRSF3. NF-κB was also shown to be involved in SRSF3-regulated miR-1908 expression (51).

### Regulation of DNA Repair

Recently, SRSF3 was identified as a regulator of the homologous recombination-mediated DNA repair (HRR) process, which may regulate the HRR-related gene expression indirectly by an epigenetic pathway (54). SRSF3 knockdown impaired HRR activity and improved the level of γ-H2AX which ating as a biomarker for double-strand DNA breaks. It also downregulated the genes involved in HRR, including *BRCA1, BRIP1*, and *RAD51*, changed the *KMT2C* (a H3K4-specific histone methyltransferase) splicing pattern, and decreased the mono- and trimethylated H3K4 level (54).

## SRSF3 Expression Regulation

**Figure 3:**
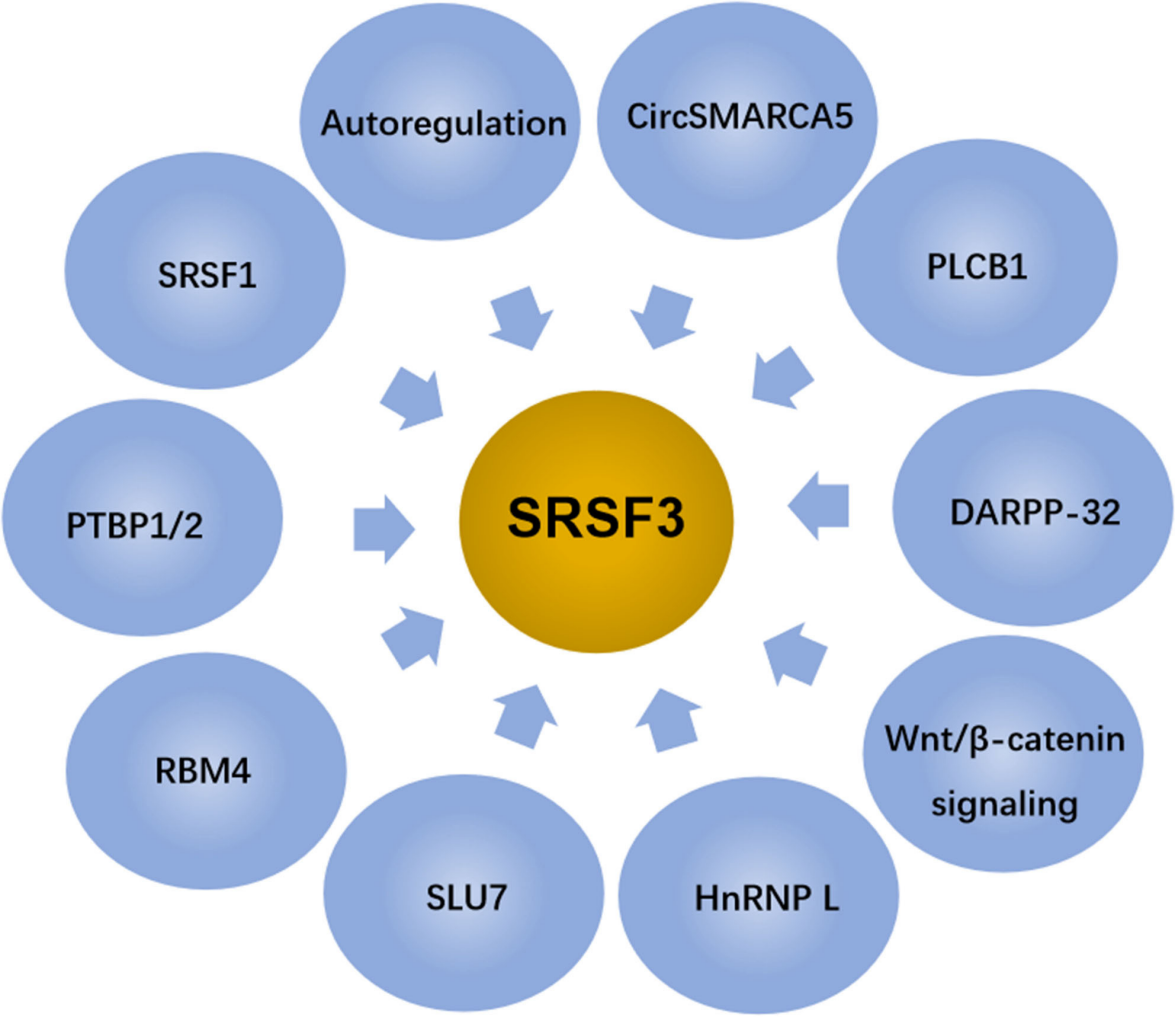
SRSF3 expression is regulated by several factors and signaling pathways.

Given the above, SRSF3 is an essential gene for embryogenesis. SRSF3 exists in oocytes and the early phase of embryonic development (19, 55), and SRSF3 missing leads to the arrest at one/two-cell developmental stage (19). In addition, it was found that the SRSF3-zygotic knockout embryos, using Cre-loxP-mediated recombination in mice to stimulate the expression of *SRSF3* gene, died at the morula stage, failing to form blastocysts (55). Contrarily, the overexpression of SRSF3 in rodent fibroblasts leads to tumorigenesis with immortal cell growth and transformation (11). Thus, the SRSF3 level in cells is controlled by multiple factors and involves complex mechanisms, as shown in Figure 3.

### Autoregulation

Autoregulation is a common mechanism for maintaining relatively stable expression of splicing factors (56). SRSF3 is the first SR protein identified as a auto-regulator for itself alternative splicing and be regulated by other members of the SR protein family (57). Genomic SRSF3 constructs are able to express two different forms of SRSF3 because of the alternative splicing of exon 4 (also referred to as intron 3 owning an in-frame stop codon), generating a full-length isoform that lacks exon 4 (*Iso1*) and an alternative isoform that includes exon 4 (*Iso2*) (57, 58). The overexpression of SRSF3 reduces the level of exon 4-skipped *SRSF3* transcripts and activates the outcome of transcripts containing exon 4 (*SRSF3-ISO2*), resulting in a truncated protein lacking the C-terminal RS domain SRSF3-TR later, which has been identified as an autoregulatory mechanism for avoiding SRSF3 accumulation (44, 57).

### Regulation by Other Splicing Factors

Besides autoregulation, SRSF3 interacts with other RNA binding proteins, including other SR splicing factors. SRSF1 (also called ASF/SF2), another member of the SR family, can lead to the disappearance of the exon 4-included isoform without affecting the amount of the skipped isoform. The outcome of the included isoform is restrained by SRSF1 even in the presence of the transfection of wild-type genomic *SRSF3*, suggesting that SRSF1 is dominant over SRSF3 in this system. These results indicate that SRSF1 and SRSF3 have adverse effects on *SRSF3* exon 4 splicing with SRSF3 playing as an activator and SRSF1 as a suppresser (57). In addition to SRSF1, other splicing factors including PTBP1 and PTBP2, can also antagonize the autoregulation of *SRSF3* splicing. PTBP1 overexpression and the presence of neuron-enriched homolog of PTBP1 and PTBP2 can shift the transcript expressions from coding *SRSF3* to *SRSF3*^+^ *exon 4*′ (59). Further study found that PTBP1 and PTBP2 can inhibit the inclusion of an exonic splicing suppressor (an ESS motif with pyrimidine-rich) by binding to it, which leads to the overexpression of full-length SRSF3 (56). In addition, RNA-binding motif protein 4 (RBM4) (an antagonizer of PTBPs), was studied to determine whether it has a antagonistic effect on *SRSF3* splicing regulation (59). Results show that RBM4 overexpression robustly shifts the transcript expressions from coding *SRSF3* to *SRSF3*^+*exon*4^′, whereas RBM4 excision inversely resulted in the increasing of the coding *SRSF3* transcript. The mutations of RNA recognition motif or substitution of the serine-to-aspartate (S309D) impedes the impact of RBM4 on productions of *SRSF3*^+*exon*4^′ transcripts, which suggests that RBM4 interferes with *SRSF3* splicing at the post-transcriptional level (59). These data indicate that there is an antagonistic effect between RBM4 and PTBPs on the utilizing of *SRSF3* exon 4.

Moreover, SLU7, a another critical splicing factor for the 3′ splice site selection (60), is also reported to modulate *SRSF3* splicing; that is, the knockdown of SLU7 induces an increase in the ratio of *SRSF3 Iso2*/*Iso1*, while SLU7 overexpression has the opposite effect (44). Further study found that SLU7 can inhibit the increasing of SRSF3-TR proteins at two levels: during the regulation of *SRSF3* splicing and during the expression of miR-17 that can target *SRSF3*-*Iso2* and promote its degradation (44).

hnRNP L is a multi-functional splicing factor that is active in a series of RNA processes, including chromatin modification, mRNA export, mRNA stability, alternative splicing, poly(A) site selection, and translational regulation (61). It was found that hnRNP L knockdown reduced the expression of SRSF3 in many cancer cell lines (62). The overexpression of hnRNP L has been found to promote SRSF3 expression in caner cells. In addition, the expression levels of hnRNP L were found to positively correlate, moderately, with the expression levels of SRSF3 in OSCC tissues. hnRNP L expression correlates with SRSF3 expression in OSCC tissues (62).

### Regulation by Wnt/β-Catenin Signaling

Wnt/β-catenin signaling is a highly conserved pathway in eukaryotic cells, its activation depends on the involvement of β-catenin in signal transduction (63). Generally, free cytoplasmic β-catenin is translocated to the nucleus to bind to the T-cell factor/lymphocyte enhancer factor (TCF/LEF), resulting in the displacement of co-repressors and recruitment of additional co-activators for Wnt target genes (64). SRSF3 is determined as a target of β-catenin/TCF4 signaling, and both the transcript and protein levels of SRSF3 are regulated by the activity of β-catenin (65–68). The isolated *SRSF3* gene promoter makes responds to influence of β-catenin/TCF4 signaling. Further study demonstrates that an increasing of SRSF3 protein levels mediated by the β-catenin/TCF4 pathway is sufficient to regulate alternative splicing decisions (67, 68).

### Regulation by DARPP-32

DARPP-32 (dopamine and cyclic adenosine monophosphate-regulated phosphoprotein, Mr 32000) is a master molecular regulator in neurons that receive the neurotransmitter dopamine (69). It was found that stable overexpression of DARPP-32 enhanced SRSF3 protein level, while endogenous DARPP-32 knockdown significantly decreased SRSF3 protein expression. Interestingly, overexpression or knockdown of endogenous DARPP-32 had no significant effects on *SRSF3* mRNA levels. Further experiments in immunoprecipitation and immunoblotting showed the co-existence of DARPP-32 and SRSF3, and DARPP-32 could prolong the SRSF3 protein half-life (20.5 h) compared with that of the control (14.9 h). Finally, DARPP-32 was proved to stabilize the SRSF3 protein by regulating its ubiquitination, which subsequently triggered the degradation of SRSF3 protein. This indicates that DARPP-32 positively regulates SRSF3 protein levels through a post-translational mechanism (70).

### Regulation by PLCβ1

PLCβ1 (phospholipase C beta 1) acts an significant role in the intracellular transduction of multiple extracellular cell signals with the assistant of calcium. An increasing evidences show that PLCβ1 is the main isoform of PLC locates in the nucleus in a phosphoinositide-specific manner (71). The overexpression of PLCβ1 causes the decrease of SRSF3 protein level. Further study found that SRSF3 could interacted with nuclear PLCβ1 at the nuclear level. These results suggest that SRSF3 is a novel target gene of the nuclear phosphoinositide-specific PLCβ1 signaling and creates new stages for the metabolism of nuclear inositol lipid (72).

### Regulation by CircSMARCA5

circSMARCA5, an exonic circRNA, was found to be in high numbers in the human brain, and was proved to act as a regulator of *SRSF3* splicing. In glioblastoma biopsies, circSMARCA5 was markedly downregulated comparing with the normal brain tissues (73, 74). circSMARCA5 overexpressing was able to increase the expression levels of *SRSF3* isoform including exon 4 (*SRSF3 Ex4*) in cells. Consistently, a significant increasing of *SRSF3* isoform without exon 4 (*SRSF3 No Ex4*) in biopsies exhibits a observably downregulation of circSMARCA5. Precipitously, *SRSF3 Ex4* levels was also upregulated in biopsies, consistent with the data obtained from the circSMARCA5 overexpression cells. This indicates that there is a positively correlation between the *SRSF3 Ex4*/S*RSF3 No Ex4* ratio and the expression levels of circSMARCA5 in glioblastoma biopsies (74).

## SRSF3 in Cancer

Despite the above-mentioned regulatory mechanisms for maintaining constant SRSF3 levels, many environmental factors can still influence the expression of SRSF3, such as human papillomavirus (HPV) (75), hepatitis B virus-encoded X protein (HBx) (76), hypoxia (29, 77), low pH (78), carcinogen DMBA (79), caffeine (80), amiodarone (81), small molecule amiloride (82), digoxin (83), and theophylline (84). Thus, the aberrant expression of SRSF3 closely relates to the occurrence, development, prognosis, and treatment response of diseases, including cancer.

### Aberrant Expression of SRSF3 in Cancer

SRSF3, as a potential diagnostic and prognostic biomarker, is upregulated in multiple types of human cancer, including breast cancer (85–88), ovarian cancer (26, 89), retinoblastoma (90, 91), head and neck cell squamous (62, 79, 92), glioblastoma (GBM) (23), gastric cancer (36), colorectal cancer (CRC) (33, 36, 93), cervical cancer (94), and hepatocellular carcinoma (HCC) (30, 95). Moreover, studies show SRSF3 upregulation not only in epithelial cancers, but also in mesenchymal tumors, as Table 1 shows (11). In addition, the single nucleotide polymorphisms (SNPs) of SFRS3 were also associated with tumor progression and prognosis. Studies report that three SNPs in SRSF3 (rs2145048, rs1406945, and rs9394364) were found in breast cancer, which may be associated with susceptibility to cancer. Among these SNPs, the C allele of rs1406945 was found to be related with increased breast cancer risk, the A allele of rs9394364 was associated with a marginally lower breast cancer risk, and the A allele of rs2145048 was associated with a lower breast cancer risk (104). Interestingly, there are contradictory reports on the expression and function of SRSF3 in colorectal cancer. It was reported that SRSF3 and hnRNPA1 indicated the two highest increasing incidences (88 and 74%, respectively), for colorectal cancer (97). There is no statistically significant correlation between the mRNA levels of *SRSF3* and the histological features, lymph node metastasis, or tumor node metastasis (TNM) stage (97). However, it was found that SRSF3 was significantly upregulated in a normal colon, but it had different expression levels (negative to strong) in colorectal cancer tissues (33, 93). SRSF3 presents a gradual expression loss during cancer progression. SRSF3 is negative or weakly positive expressed in 80% patients with metastatic stage IV, which was markedly related to poor survival in colorectal cancer (93). Similar to colorectal cancer, the expression and function of SRSF3 are also contradictory in liver diseases. In mice, SRSF3 overexpression was proved to be crucial for maintaining hepatocyte metabolic function and differentiation (20, 105). The deletion of SRSF3 damages the maturation and metabolism of hepatocyte during early adulthood in mice developed spontaneous HCC as they aged. In addition, SRSF3 may play a role in the prevention of hepatic carcinogenesis by regulating splicing to inhibit fibrosis, mitogenic splicing, and epithelial-mesenchymal transition (EMT) (105). In line with these results, hepatic SRSF3 expression was decreased in mouse models of non-alcoholic fatty liver disease (NAFLD) and non-alcoholic steatohepatitis (NASH). Thus, the avoidance of SRSF3 degradation in mice can protect them from hepatic steatosis, fibrosis, and inflammation, to some extent (98). However, SRSF3 expression presented no changes in mouse models of PTEN-deficient HCC and DEN-induced HCC (95). In human HCC, it was reported that SRSF3 expression was either downregulated or the protein was mislocalized (105), whereas Wang found a significant increasing expression of SRSF3 in human HCC tumors (30, 95), which emerged progressive upregulation from a normal liver to a cirrhosis/fibrosis liver, and ultimately HCC (30). In addition, upregulation of isoforms *SRSF3* was also found in these tissues (30, 95), which might enhance the development of HCC by regulating splicing of SRSF3 targets (30). Consequently, it is likely that SRSF3 presents low expression and tumor-suppressor activity in mouse liver disease, while it shows high expression and acts as an oncogene in human HCC, suggesting its role as an unfavorable prognostic predictor in HCC. Unexpectedly, there is a positive links between SRSF3 upregulation and longer overall survival in patients with HCC resection (95).

**Table 1:** Clinical features, cell functions and related genes associated with upregulation of SRSF3 in human cancer.

**Tumor type**	**Expression**	**Role**	**Clinic relevance**	**Cell line**	**Function**	**Related gene**	**References**
Glioblastoma	Upregulated	Oncogene	High tumor grades	U87, GSC83, GSC528, GSC23	Tumorigenicity, proliferation, cell growth, self-renewal	ETV1, NDE1	(23)
Ovarian cancer	Upregulated	Oncogene	Poor clinical parameters, poor survival	A2780, IGROV1, SKOV3	Drug resistance, apoptosis, metastasis	Bcl-2, MRP1, CD44	(26, 89, 96)
Colorectal cancer	Upregulated or gradual loss	Oncogene	No significance (based on protein level); advanced cancer progression, poor survival (based on protein level)	HCT-116, HT-29, KM12C, CaCo-2, HCT-8, Colo205, HCT-116, DLD-1, WiDr, KM12SM, SW480	Tumorigenicity, proliferation, metastasis, adhesion, invasion, apoptosis	RBM4, MAP4K4, JNK1, E-cadherin, N-cadherin, β-catenin, MCC, PKM, E2F1/7, vimentin, cyclins (D1/D3/E1), HIPK2, Bcl-2	(11, 28, 33, 36, 39, 59, 65, 93, 97)
Hepatocellular carcinoma	Upregulated	Oncogene	Positive associated with HBV-associated HCC in patients with higher AFP levels; poor overall survival; longer overall survival in patients with HCC resection	Bel-7404, HepG2, Bel-7402, MHCC97H, MHCC97L, HepG2.2.15	Colony formation; proliferation, migration; invasion	NEDD8, ARHGEF2, 14-3-3β, Ras, Foxo4, CCDC50S, HBX	(11, 30, 76, 95, 98)
Osteosarcoma	Upregulated	Oncogene	Unknown	U2OS, Rh30	Colony formation, transformation, proliferation, migration, invasion cell cycle, apoptosis	CCND1, miR-1908-5p, REST, miR-132-3p, miR-212-3p, YAP1, NF-κB, NKIRAS2, IL-3; PDCD4	(11, 36, 39, 52, 99, 100)
Breast cancer	Upregulated	Oncogene	High tumor grade, poor tumor progression and prognosis	MDA-MB231, SKBR3	Transformation, proliferation, apoptosis, metastasis, cell cycle	p53; TDP4; PAR3; NUMB; HER2	(11, 84–88, 101)
Gastric cancer	Upregulated	Oncogene	No significance				(11, 97)
Cervical cancer	Upregulation	Oncogene	Increased diagnostic accuracies comparable to CEA	Hela, C33A, CaSki	Colony formation, proliferation, apoptosis, migration, invasion, cell cycle	p53, REST, miR-132-3p, PLK1, CCND1, FoxM1, miR-212-3p, YAP1, Cdc25B, IL-3	(11, 84, 94, 99, 100)
Head and neck squamous cell carcinoma	Upregulated	Oncogene	high tumor grade, worse lymphatic metastasis, poor survival	CAL27, FaDu, SCC-9	Autophagy, cell growth	BECN1, FoxO1, Snail, p65, N-cadheri, hnRNP L	(62, 79, 92, 102)
Retinoblastoma	Upregulated	Oncogene	Unknown	Unknown	Unknown	Unknown	(90, 91)
Others cancers (lung, bladder, kidney, skin, thyroid)	Upregulated	Oncogene	Unknown	JSC-1, BCBL1, SUDHL-6, T24, 253J-BV, PC3, KATOIII, A375, NUGC3, MKN7	proliferation, apoptosis	MDM2/4, p21, BBC3	(11, 24, 33)
Acute myeloid leukemia	Downregulated	Oncogene	Unknown	Unknown	Unknown	Caspase-8	(98)
Renal cancer	Downregulated	Oncogene	Unknown	786-O	Proliferation	Unknown	(11, 103)

In contrast to the upregulation of SRSF3 in the above tumors, the expression of SRSF3 mRNAs was significantly decreased in acute myeloid leukemia (103) and renal cancer (106), as shown in Table 1. However, it was also identified as an oncogene in these two types of cancers. Nonetheless, the correlation between SRSF3 expression and carcinogenesis and the progression of these cancers, such as histological features, lymph node metastasis, TNM stage, or overall survival, remains to be studied.

#### Pro-oncogenic Activity of SRSF3 by Regulating Cellular Biological Processes

**Figure 4:**
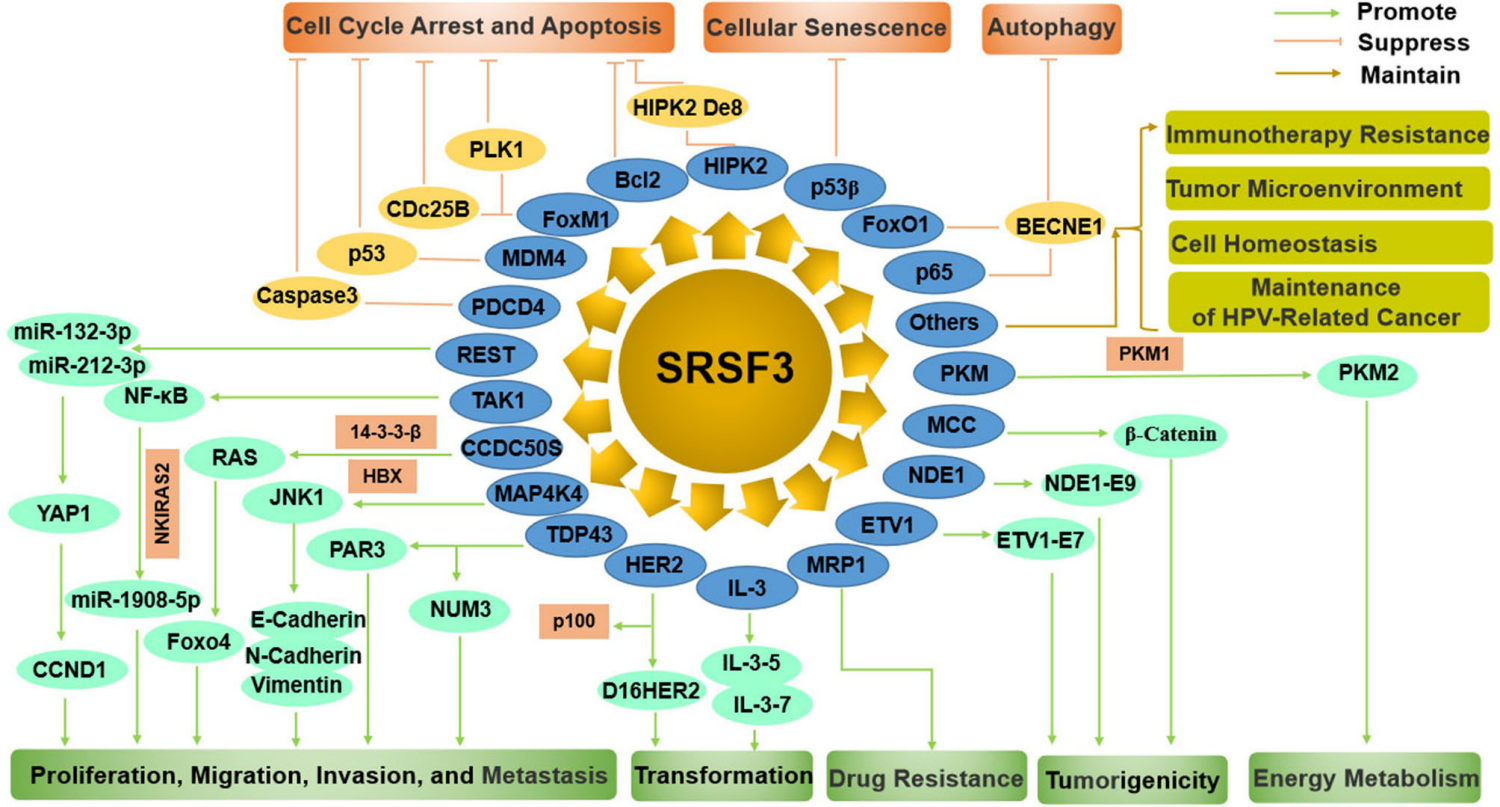
The comprehensive mechanism of SRSF3 functions as an oncogene by regulating multiple splicing targets in cancer cells. On the one hand, SRSF3 can enhance cell proliferation, migration, invasion, metastasis, transformation, drug resistance, tumorigenesis, and energy metabolism. On the other hand, SRSF3 can inhibit cell cycle arrest, apoptosis, cellular senescence, and autophagy. SRSF3 is also involved in immunotherapy resistance, tumor microenvironment, cell homeostasis, and maintenance of HPV-related cancer.

SRSF3 functions as an oncogene manipulating cell proliferation, cell cycle, apoptosis, migration, invasion, transformation, tumorigenesis, metastasis, drug resistance, autophagy, and cellular senescence by regulating many pathways, including p53, JNK, Ras, Wnt, HER2 signaling pathways, and miRNAs (Figure 4).

#### Enhancement of Cell Proliferation, Migration, Invasion, and Metastasis

SRSF3-silencing inhibits the proliferation, migration, invasion, and metastasis of tumor cells (33, 39, 52, 99). It was reported that SRSF3 affected the expression levels of miR-132-3p and miR-212-3p, including both their primary form and their mature form, by controlling RE1-silencing transcription factor (REST) in cancer cells (99). miR-132-3p and miR-212-3p were found to inhibit the malignant phenotypes of cancer cells by repressing Yes-associated protein 1 (YAP1) and its downstream gene CCND1 (Cyclin D1), which demonstrates that SRSF3 gives malignant characteristics to cancer cells by SRSF3/REST/miR-132-3p (miR-212-3p)/YAP1/CCND1 axis (99). Beside miR-132-3p and miR-212-3p, SRSF3 may also upregulate the expression of miR-1908-5p by enhancing NF-κB transactivation (52). Interestingly, miR-1908-5p in turn could downregulate NF-κB activation by binding to an inhibition factor of NF-κB pathway, NF-κB inhibitor interacting Ras-like 2 (NKIRAS2), resulting in elevating cancer cell proliferation and metastatic potential (52). These data suggest that SRSF3 enhances the malignant characterization of cancer cells via the SRSF3/miRNA axis.

CCDC50, as a tyrosine-phosphorylated protein, is required for cell survival as it inhibits the NF-κB or p53 mediated apoptotic pathway (107, 108). *CCDC50S*, as a truncated oncogenic splice variant, was highly expressed in HCC and significantly correlated with progression and predicts poor survival of patients (76). SRSF3 was reported to directly bind to *CCDC50S* mRNA for its maintenance in the cytoplasm, resulting in the promotion of HCC progression by increased activation of Ras/forkhead box protein O4 (Foxo4) signaling (76). Contrarily, SRSF3 was mediated by the interaction of HBx and 14-3-3β, which demonstrated the existence of the HBx/14-3-3β/SRSF3/CCDC50S/Foxo4 axis in oncogenic progression of HCC (76).

Mitogen-activated protein 4 kinase 4 (MAP4K4) belongs to the STE20/MAP4K family that content a serine/threonine kinase domain, and is involved in cytoskeletal rearrangement and migration by regulating the MAPK/ERK kinase cascade (109). It was reported that alternatively spliced *MAP4K4* variants showed differential influences on the EMT process, which is a critical process for the migration and invasion of cancer cells (59). SRSF3 could modulate the usage of *MAP4K4* exon 16′ in a sequence specific manner, while the inclusion of *SRSF3* exon 4′could be enhanced by RBM4 in colorectal cancer cells (59). These date suggest that the RBM4-SRSF3-MAP4K4 axis manipulate the metastasis of cancer cells through the EMT process.

TDP43 (TAR DNA-binding protein), is a highly conserved and an important splicing regulator that controls gene expression. TDP43, as a major splicing regulator in triple-negative breast cancer (TNBC), is associated with poor prognosis in TNBC progression (86). And TDP43 overexpression could significantly enhance the proliferation and malignancy of mammary epithelial cell (86). In coordination with SRSF3, TDP43 can alter most splicing events, including *PAR3* and *NUMB* which play essential roles in cell proliferation and metastasis (86). Further study found that the TDP43/SRSF3/PAR3 axis regulated the metastasis of cancer cells, while the TDP43/SRSF3/NUMB axis controlled the proliferation of cancer cells (86).

#### Enhancement of Cell Transformation

HER2 (ErbB2) is a member of the erythroblastic oncogene B (ErbB) family of receptor tyrosine kinases. The overexpression of HER2 is associated with many aggressive tumors and a poor prognosis (110). *HER2* possesses several splice variants that produce diverse proteins with various biological activities and functions in tumor development (111). SRSF3 was identified as an important splicing regulator of HER2. Loss of SRSF3 results in alterations in all splice variants of *HER2*. Especially, SRSF3 knockdown leads to a switch in *HER2* mRNA splice variants from Δ*16HER2* to *p100* (101). Interestingly, the function of these two splice variants is contradictory. Δ*16HER2* is a highly tumorigenic factor that is likely to increase malignant transformation of breast cancer cells (112, 113), whereas *p100* is involved in the inhibition of tumor cell proliferation and oncogenic signals (114). That is, the overexpression of SRSF3 in tumors switches splicing variants of *HER2* mRNA from *p100* to Δ*16HER2*, leading to tumor progression (101).

Moreover, SRSF3 also controls the production of various splicing variants of interleukin enhancer binding factor 3 (ILF3) by the exclusion, inclusion, or 3' splice site selection of *ILF3* exon 18 (100). SRSF3 knockdown expression produced aberrant isoform-5 and−7 of *ILF3* in osteosarcoma cancer cells. Both isoform-5 and−7 can inhibit tumor cell proliferation, and isoform-7 can also induce cell apoptosis. SRSF3 overexpression in cancer cells has a positive association with the steady status maintenance of *ILF3* isoform-1 and−2, which can promote cell proliferation and transformation (100).

#### Enhancement of Drug Resistance

The main obstacle to the improvement of prognosis is cancer chemotherapy resistance. The multidrug resistance protein 1 (MRP1) belongs to the ATP-binding cassette transporter subfamily linked to multidrug resistance (115, 116). MRP1 upregulation may trigger resistance to various chemotherapeutic drugs in ovarian cancer cell lines. MRP1 has also been reported to be involved in clinical drug resistance and to be of prognostic significance for predicting patients' response to chemotherapy (117–119). Interestingly, more *MRP1* mRNA splice variants were found in ovarian tumors compared to the matched normal tissues (26). These variants can confer drug resistance even if they are not as effective as the full-length *MRP1*. Further study found that SRSF3 overexpressed in ovarian tumors could result in more splicing variants of *MRP1* mRNA by increasing the identification of weak exons (26), which indicates that SRSF3 may be involved in the cancer chemotherapy resistance.

#### Enhancement of Cell Tumorigenicity

In patient-derived glioma stem-like cells (GSC), the increasing expression of SRSF3 causes the significantly improvement of cell proliferation, self-renewal, and tumorigenesis (23). More than 1,000 SRSF3-related AS events are identified by transcriptomic profiling, and they have a preference for exon skipping in cell mitosis genes. SRSF3 knockout results in the exon skipping at exon 7 of transcription factor ETS variant 1 (*ETV1*) to product *ETV1-E7*, leading to the enhancement of the proliferation and sphere formation ability of tumor cells. Further, the phosphorylation of *ETV1-E7*-encoded peptide could enhance the oncogenic activity of ETV1, promoting an ETV1-mediated oncogenic transcriptional program in GSCs. Moreover, SRSF3 knockout also induced the nudE neurodevelopment protein 1 (*NDE1*) gene to a mutually exclusive exon 9' taking the place of the terminal exon 9, resulting in the production of isoform-specific function of *NDE1 (NDE1-E9)* in mitotic spindle formation that is important for tumor cell growth (23).

Similarly, in CD133^+^ colon cancer stem like cells (CSLCs), SRSF3 was overexpressed and acted a part in the oncogenicity of colon CSLCs by regulation of the Wnt/b-catenin pathway (65). SRSF3, as a novel target of the Wnt/b-catenin pathway, was upregulated by Wnt pathway activation in CD133^+^ colon cancer cells (68). SRSF3 exerts a powerful negative effect on the expression of the mutated in colorectal cancer (MCC) protein expression, which is significantly upregulated in various CRC cell lines. Interestingly, the MCC protein could interact with β-catenin, resulting in the inhibition of Wnt signaling (65, 120), suggesting that SRSF3 may be involved in the Wnt pathway modulation, resulting in forming positive feedback relationships among the Wnt/b-catenin pathway, SRSF3, and MCC.

#### Enhancement of Energy Metabolism

Alternative splicing of the pyruvate kinase M (PKM) can produce the pyruvate kinase muscle 2 (PKM2) isoform and promote aerobic glycolysis and tumor growth (121). PKM is controlled by mutually beneficial effects on the two mutually exclusive exons 9 and 10 in cancer cells, resulting in the repression of exon 9 and the activation of exon 10. SRSF3 was found to activate exon 10, mediating changes in glucose metabolism (22). Loss of SRSF3 in human colon cancer cells induces an increasing in the ratio of PKM1/PKM2, leading to a metabolic shift from glycolysis toward oxidative phosphorylation. Moreover, the SRSF3 silenced cells causes markedly inhibition of cell growth and autophagy (33). These findings indicate that SRSF3 acts as a critically positive regulator for *PKM* mRNA splicing and cancer-specific energy metabolism.

#### Inhibition of Cell Cycle Arrest and Apoptosis

A decreased level of SRSF3 could induce cell apoptosis and reduce cell proliferation in SW480 (human colon adenocarcinoma) and U2OS (human osteosarcoma) cells (39). Microarray analyses shows that SRSF3 silencing causes the upregulation of 381 genes and the downregulation of 274 genes in U2OS cells. Among them, A number of genes are related to the apoptotic and anti-apoptotic processes, including *PDCD4*, who was the most affected (39). PDCD4 was reported to be a tumor suppressor that was involved in cellular processes, such as antiproliferation, apoptosis, and antimetastasis in various cancer cells (122, 123). As mentioned above (functions of SRSF3), SRSF3 was further proved to participate in alternative splicing and the export and translation of *PDCD4* mRNA, leading to the downregulation of the PDCD4 protein (36, 39). SRSF3 and PDCD4 knockdown could prevent tumor cell apoptosis with decreased Caspase-3 activation and decreased amount of fragmented chromosomal DNA. These results indicate that the effects of PDCD4 on cell proliferation and apoptosis might be dependent on the expression levels of PDCD4 (39).

The p53 tumor suppressor gene is a nuclear transcription factor that transmits signals arising from many types of genotoxic or cellular stress, such as DNA damage, hypoxia, and nucleotide deprivation, to the target genes and related factors that induce cell cycle arrest, cell death, or cellular senescence (124). The tumor-suppressor function of p53 can be induced or inhibited by many other genes, including SRSF3. Reportedly, the inactivation of SRSF3 with the inclusion of *MDM4* exon 6, can stimulate p53 activation (24). SRSF3 was found to be necessary for *MDM4* exon 6 inclusion and the growth of melanoma. In embryonic tissues and cancers, the enhancement of exon 6 inclusion can significantly upregulate the levels of MDM4 protein to inhibit the tumor-suppressor function of p53 (125, 126). In human cancers, an increasing expression of MDM4 is promoted by a non-sense-mediated, decay-targeted isoform of *MDM4 (MDM4-S)* by enhancing exon 6 inclusion (127, 128). The knockdown of SRSF3 leads to MDM4 downregulation, resulting in the activation of p53 pathway as well as its target genes, such as *p21, MDM2*, and *BBC3* (24). Consequently, cell growth is markedly decreased, while cell death is increased in SRSF3 silencing cells.

The increased expression of SRSF3 overexpression in cancer cells causes the improvement of cell cycle performance by influencing the expression levels of G2/M transition regulators, including Forkhead box transcription factor M1 (FoxM1), PLK1, and Cdc25B. Conversely, SRSF3 silencing causes G2/M arrest, growth retardation, and apoptosis (11). Further study documented that SRSF3 silencing also caused G1 arrest in combination with the downregulation of several G1/S checkpoint regulators, including cyclins (D1, D3, and E1), E2F1, and E2F7, which likely impaired G1-to-S-phase progression (28). In addition, SRSF3 silencing could induce cell apoptosis by reduction of Bcl-2 (28, 96). Moreover, SRSF3 silencing changed the alternative splicing of homeodomain-interacting protein kinase2 (HIPK2), resulting in the production of *HIPK2 De8* isoform which facilitated the cell apoptosis by phosphorylating p53 at Ser46 (28). These results expose the critical role of SRSF3 in the regulation of G1-S and G2-M cycle performance, Bcl-2 expression, and HIPK2-mediated cell apoptosis (28).

#### Cellular Senescence Inhibition

Cellular senescence, an irreversible proliferation arrest, is identified as another endogenous mechanism that represses tumorigenesis in company with cell death programs (129–131). Endogenous SRSF3 knockdown could induce cellular senescence, and upregulate the expression of *p53*β (an alternatively spliced isoform of *p53*) to trigger p53-mediated senescence (27). p53 silencing restores SRSF3-knockdown-induced cellular senescence in part, suggesting that SRSF3 plays a role in the initiation of p53-mediated cellular senescence. Further, SRSF3 was found to bind to an alternatively-spliced exon of *p53*β mRNA in sequence-dependent manner. (27), suggesting that SRSF3 is an inhibitor in p53-mediated cellular senescence.

#### Inhibition of Autophagy

Autophagy is an evolutionarily conserved cellular catabolic process. SRSF3 can act as an autophagy suppressor (33, 102). SRSF3 knockdown significantly induces autophagy with an increased LC3B-II/LC3B-I ratio, whereas the overexpression of SRSF3 inhibits autophagy induction with an decreased ratio of LC3B-II/LC3B-I. Moreover, SRSF3 knockdown plus autophagic degradation inhibitor chloroquine could enhance the accumulation of LC3B-II, suggesting that SRSF3 knockdown truly increases autophagic flux. The molecular mechanism is due to the suppression of the FoxO1 and p65 expressions as well as the transcriptional and protein levels of BECN1 (102).

#### Others

SRSF3-mediated alternative splicing was also reported to be associated with many other key genes, such as *CD19* (132), *CD44* (68, 133), and *VEGF* (78), and to be involved in other tumor related biology process, including the resistance to CART-19 immunotherapy (132), tumor cell homeostasis (21), tumor microenvironment (low pH) (78), and the maintenance of HPV-related cancer (134). With the growing knowledge on SRSF3 overexpression or knockout in human tissues and cells by high-throughput RNA-sequencing, more possible target genes and underlying mechanisms will be elucidated. It is even possible that the new data on cancer cells might differ from that on non-cancer cells, reflecting the oncogenic effects *vs*. tumor suppressor effects when expressed at high *vs*. low levels of SRSF3. Nevertheless, considerable efforts and in-depth studies are expected to provide more information on SRSF3 and their targets.

#### Implications for Therapy

**Figure 5:**
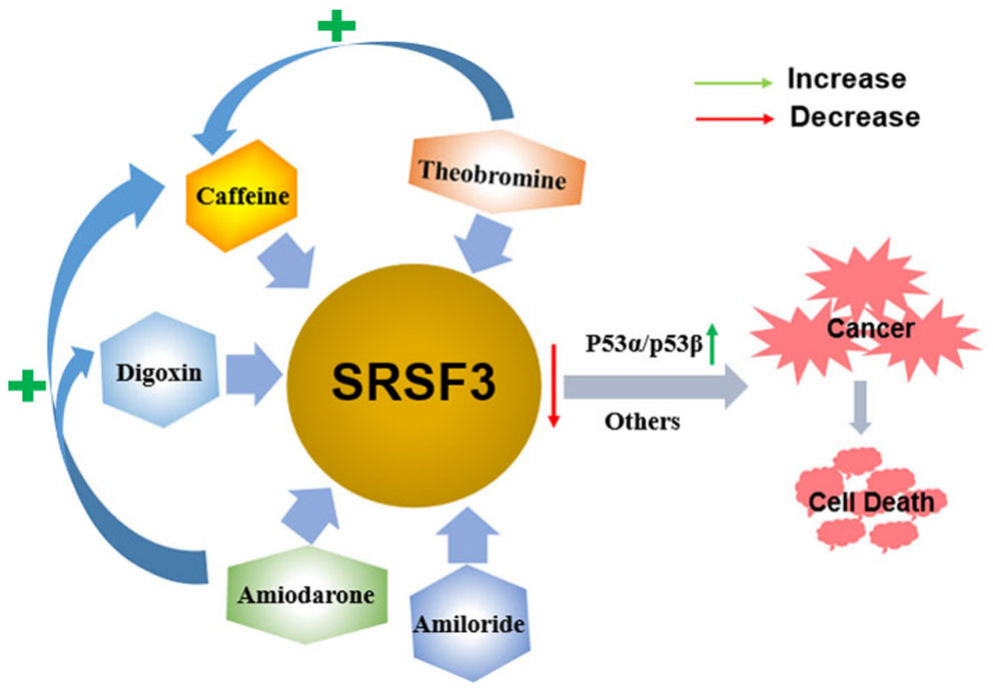
The antitumor function of some drugs via decreasing the expression of SRSF3. Caffeine, digoxin, amiodarone, amiloride, and theobromine induce the apoptosis of cancer cells by decreasing the expression of SRSF3 and its downstream signaling cascade, including inducing the switch of *p53* splicing from *p53*α to the *p53*β isoform. Moreover, theophylline and amiodarone could enhance caffeine-induced cell death, and amiodarone could also enhance the efficacy of digoxin.

The multifunctional characteristics of SRSF3 highlight it as a novel splicing regulator for gene expression and cell homeostasis. Given the crucial roles of alternative splicing in cancer biology, pharmacological modulation of SRSF3-mediated splicing may represent an important therapeutic strategy. Indeed, thus far, SRSF3 has been evidenced to associate with the antitumor function of some drugs in the development of targeted therapeutics for the treatment of cancer, as shown in Figure 5.

Caffeine, a 1,3,7-trimethylxanthine derivative, is a potential anticancer drug that inhibits cell proliferation and induces apoptosis on various cancer cells *in vitro* and *in vivo* (135–137). The alternative splicing of cancer-related genes is involved in caffeine-induced antitumor function, including p53, PKM2, and hypoxia-inducible factor-1α/2α (HIF-1α/2α) (80, 138–140). In cervical cancer cells, caffeine regulates cell-cycle arrest and cell apoptosis by decreasing SRSF3 expression that modifies the expression of various splice variants of *p53*, including reducing *p53*α expression and inducing *p53*β expression. In addition to p53-dependent functions, multiple genes involved in the EMT and hypoxic conditions are all found to be regulated by SRSF3 (80). Theobromine (1,3-dimethylxanthine), a metabolite of caffeine, may also downregulate SRSF3 expression by switching *p53* from *p53*α into *p53*β, which is similar to caffeine. Consequently, theophylline demonstrates antitumor roles via inducing cellular apoptosis, senescence, and decreasing colony formation. Moreover, theophylline could synergistically enhance caffeine-induced cell death (84). A similar switch of *p53* splicing from *p53*α to *p53*β is induced by digoxin, a popular cardiac glycoside identified as a potential anticancer drug (83, 141). Similar to caffeine, digoxin regulates G2/M arrest, DNA damage, and cell apoptosis via p53-dependent pathway in cervical cancer cells by reducing both SRSF3 expression and increasing expression of *p53*β isoform (83).

Amiodarone is an anti-arrhythmic drug commonly used to block several types of myocardial potassium channels in arrhythmia and atrial fibrillation (142, 143). Amiodarone also sensitizes tumor cells in response to chemotherapy (144, 145). It has been proved that the mechanism of action of amiodarone may be similar to that of caffeine or digoxin, it also regulates senescence through the SRSF3-p53 pathway (20, 27, 146, 147). Amiodarone could induce cellular reactive oxygen species (ROS) and suppressed cell survival in cervical cancer cells. Moreover, amiodarone is found to strengthen the effectiveness of caffeine and digoxin on cell toxicity (81). Amiodarone also reduces the SRSF3 gene and protein expression. However, it accumulates the population of SRSF3-PTC without the switch of *p53* splicing from *p53*α to *p53*β via the SRSF3 downregulation, suggesting that amiodarone causes cancer cell death in a p53-independent manner. Interestingly, amiodarone can work in coordination with caffeine and digoxin on the expression of *p53* alternative splicing isoforms from *p53*α into *p53*β via decreasing SRSF3 (81). Amiloride, 3,5-diamino-6-chloro-N-(diaminomethylene) pyrazinecarboxamide monohydrochloride, is a prototype intracellular pH (pHi) modulator medicine widely used for clinically treating in edema and hypertension depending on its sodium transport and humoral steady-state effects (148). In many solid tumor and leukemic cells in humans, amiloride was discovered to present an antitumor ability that decreased cell migration and invasion, arrested cell cycle, enhanced apoptosis, and caused severe DNA damage, and, ultimately, cell death (82). Mechanically, amiloride was proved to “normalize” the mRNA splicing of *BCL-X, HIPK3*, and *RON/MISTR1* by the decreased expression of SRSF3 and some other SR proteins in human HCC cells. Further, it was found that amiloride regulated SRs by downregulating kinases and upregulating phosphatases involved in phosphorylation pathways of SRs (82). However, further study is required to investigate whether the various antitumor drugs mentioned above regulate SRSF3 in a direct or indirect fashion.

## Discussion

**Figure 6:**
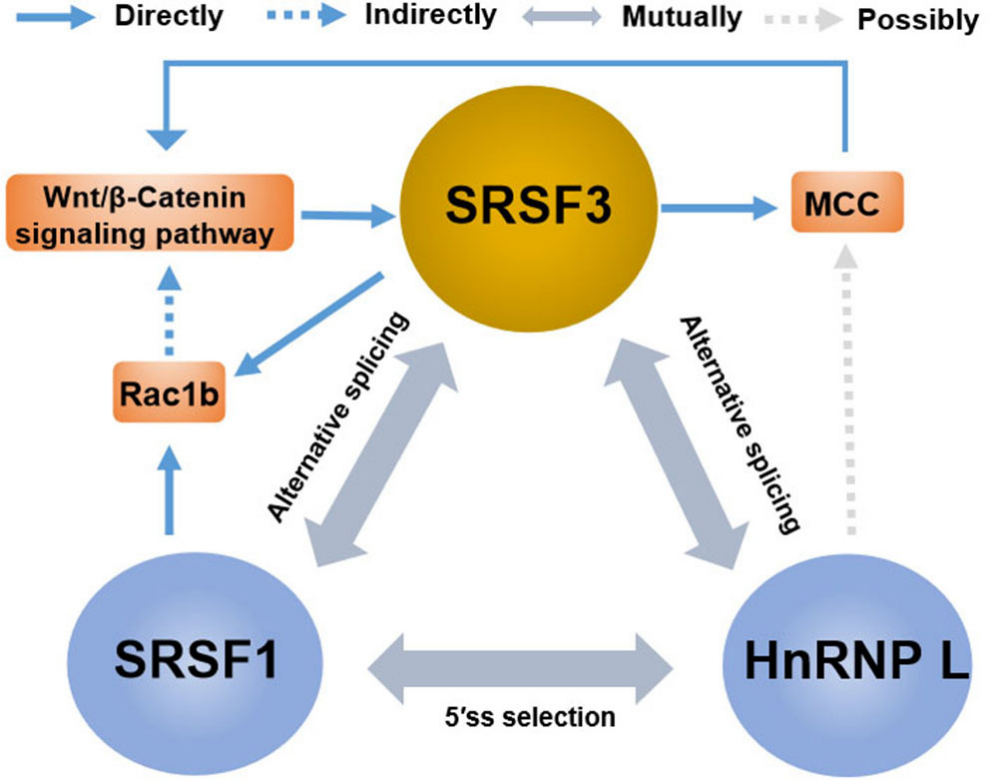
The crosstalk between SRSF3 and other splicing regulators in the regulation of gene expression. There are antagonistic effects among SRSF3, SRSF1, and HnRNP L on alternative splicing or 5'ss selection, involving the key genes or signaling pathways, such as the Wnt/β-catenin pathway, MCC protein, and Rac1b.

The rising role of alternative splicing and splicing-related factors in cancer has opened doors not only for the understanding of tumor occurrence and progression but also for the development of new targeted therapy. In reality, splicing-related factors can act either as survival-promoting factor that reduce drug-induced apoptosis or, contrarily, as potentiating the pro-apoptotic effects of chemotherapeutics (149). SRSF3 can be considered as a potential molecular switch that regulates many biological processes in cancer cells, enabling sensitization of cancer cells to therapeutic treatments. Notably, the contribution of SRSF3 to the regulation of key genes goes far beyond the splicing reaction and involves all aspects of gene expression, while also cooperating with other splicing regulators. For instance (Figure 6), in addition to the induction of the MCC protein, β-catenin/TCF4-induced SRSF3 expression decreased Rac1b expression in colorectal tumor cells by increasing skipping of alternative exon 3b (67). Interestingly, Rac1b, an alternative splicing variant of Rac1, is an oncoprotein increased in the subgroup of colorectal tumors and is necessary to maintain the viability of tumor cells (150, 151). These results may support the view that SRSF3 causes the overall change of gene expression to maintain cell homeostasis (21). This may also explain the fact that SRSF3 presents a high expression in normal colons, and the loss of SRSF3 expression is significantly correlated with low survival rate and short disease-free survival time, especially in the early step of colorectal cancer (93). Moreover, in contrast to SRSF3 acting as a silencer of endogenous Rac1b splicing, SRSF1 was found to increase the inclusion of alternative exon 3b, acting as an enhancer of *Rac1b* splicing (67). Meaning, SRSF1 and SRSF3 exhibits antagonistic effects on alternative splicing of *Rac1b*, which is in accordance with that of SRSF1 and SRSF3 having antagonistic effects on mutual splicing events (57). In addition to SRSF1, hnRNP L is the other splicing factor whose alternative splicing is regulated by SRSF3, while hnRNP L also regulates the expression of oncogene SRSF3 (62, 92). Contrarily, hnRNP L and SRSF1 also have an antagonistic effect on 5′SS selection (152). This indicates that multiple SR members share target genes and the redundancy of functions of multiple SR proteins. This also indicates that there is compensatory regulation of expression in multiple SR protein members, which is very relevant to the effectiveness of multiple drugs on SRSF3 action as there might be compensatory upregulation of other SR protein members after SRSF3 inhibition. Moreover, some RNA modification regulators are also involved in the regulation network of splicing factors, including the *N*6-methyladenosine (m6A)-binding protein YTHDC1 (YTH domain containing 1). YTHDC1 can promote exon inclusion of targeted mRNA by recruiting SRSF3 while blocking SRSF10, which expands the potential utility of m6A modification mRNA (153, 154). In a word, the mutual regulation of splicing factors is a complex and important process for expression in target genes. Thus, future study is needed for the exploration of the relationship between SRSF3 and other mRNA related factors, including that of other spliceosome-associated proteins, splicing regulatory factors, and transcriptional factors.

In terms of the importance of splicing factors in cancer pathology, emerging studies focus on the discovery of new small compounds in tumor suppression, targeting spliceosome elements (149). SRSF3 downregulation is associated with cell death by the treatment of caffeine, digoxin, theophylline, amiodarone, and amiloride in cancer cells (80–83). In addition, small inhibitors of kinases have been identified as potential chemotherapeutics to angiogenesis, including BE-13793C (155), TG003 (156), and SRPIN340 (157); it will be interesting to evaluate their impact on SRSF3. Similar to other SR proteins, SRSF3 is phosphorylated by kinases including topoisomerase I, the SR protein kinase (SRPK) family, and the CDC2-like kinase (CLK) family (24, 158–160), with affecting SR protein subcellular localization, binding to substrate mRNA and interacting with other proteins (161). Additionally, indole derivatives, such as benzopyridoindoles and pyridocarbazoles, is a recently discovered class of compounds that regulate splicing by altering the splicing activity of SR protein in company with the exonic splicing enhancer (ESE) (162). Indole derivatives have been proved to regulate splicing events that reversing the pro-metastatic splicing of Ron proto-oncogene mRNA (4, 163). Of course, the effects of SRSF3 in the functions of indole derivatives in tumor cells need extensive analysis. Nevertheless, investigating the molecular mechanisms governing SRSF3-dependent signaling will promisingly reveal new drug candidates and therapeutic targets for cancer treatment.

## Author Contributions

All authors contributed to the search of the literature and the writing of the manuscript, read, and approved the final manuscript.

## Conflict of Interest

The authors declare that the research was conducted in the absence of any commercial or financial relationships that could be construed as a potential conflict of interest.

## Abbreviations

ABC: ATP-binding cassette
BP: Branch point
AS: Alternative splicing
CLK: CDC2-like kinase
CRC: Colorectal cancer
CTD: C-terminal domain
DARPP-32, Dopamine and cyclic adenosine monophosphate-regulated phosphoprotein: Mr 32000
DNA: Deoxyribonucleic acid
ESE: Exonic splicing enhancer
FL: Follicular lymphoma
FoxM1: Forkhead box transcription factor M1
GBM: Glioblastoma
GSC: Glioma stem-like cells
GV: Germinal vesicle
GVBD: Germinal vesicle breakdown
HBx: Hepatitis B virus-encoded X protein
HCC: Hepatocellular carcinoma
HIPK2 FL: Full-length HIPK2
HIPK2: Homeodomain-interacting protein kinase2
hnRNPs: Heterogeneous nuclear ribonucleoproteins
HPV: Human papillomavirus
HRR: Homologous recombination repair
IGC: Interchromatin granule cluster
IR: Intron retention
IRES: Internal ribosome entry site
JNK: c-Jun N-terminal protein kinase
MAP4K4: Mitogen-activated protein 4 kinase 4
MCC: Mutated in colorectal cancer
miRNA: MicroRNA
mRNA: Messenger RNA
MRP1: Multidrug resistance protein 1
NAFLD: Nonalcoholic fatty liver disease
NASH: Non-alcoholic steatohepatitis
NRS: Negative regulator of splicing
NXF1: Nuclear export factor 1
NXF1-NXT1: NXF1-nuclear transport factor 2-related export protein 1
PBs: P-bodys
PDCD4: Programmed cell death
PKM: Pyruvate kinase M
PKM2: Pyruvate kinase muscle 2
PLC: Phospholipase C
pre-mRNA: Precursor messenger RNA
pri-miRNAs: Primary microRNA transcripts
PTB: Polypyrimidine tract-binding protein
PTC: Premature termination codon
RBM4: RNA-binding motif protein 4
RBPs: RNA-binding proteins
RNA: Ribonucleic acid
ROS: Reactive oxygen species
RI: Retained intron
RRM: RNA-recognition motif
RS: Arginine (R) and Serine (S)
RSV: Rous sarcoma virus
SCC: Sister chromatid cohesion
SELEX: Systematic evolution of ligands by exponential enrichment
snRNPs: Small nuclear ribonucleoproteins
SR: Ser/Arg-rich
SRE: Splicing regulatory element
SRPK: SR protein kinase
SRSF: Ser/Arg-rich Splicing Factor
TL: Terminal loop
TNBC: Triple-negative breast cancer
TNM: Tumor node metastasis
UTR: Untranslated region
YTHDC1: YTH domain containing 1.
